# ROS-responsive PPGF nanofiber membrane as a drug delivery system for long-term drug release in attenuation of osteoarthritis

**DOI:** 10.1038/s41536-022-00254-3

**Published:** 2022-11-03

**Authors:** Jianjun Wu, Zainen Qin, Xianfang Jiang, Depeng Fang, Zhenhui Lu, Li Zheng, Jinmin Zhao

**Affiliations:** 1 Guangxi Engineering Center in Biomedical Materials for Tissue and Organ Regeneration, The First Affiliated Hospital of Guangxi Medical University, Nanning, 530021 P.R. China; 2 Guangxi Collaborative Innovation Center for Biomedicine, The First Affiliated Hospital of Guangxi Medical University, Nanning, 530021 P.R. China; 3 Department of Oral Radiology, Guangxi Medical University College of Stomatology, Nanning, 530021 P.R. China; 4 Department of Orthopedics Trauma and Hand Surgery, The First Affiliated Hospital of Guangxi Medical University, Nanning, 530021 P.R. China; 5 Guangxi Key Liboratory of Regenerative Medicine, The First Affiliated Hospital of Guangxi Medical University, Nanning, 530021 China

**Keywords:** Regenerative medicine, Drug delivery

## Abstract

Excessive reactive oxygen species (ROS) are one of the leading mechanisms in the initiation and development of osteoarthritis (OA). However, conventional injection of ROS-responsive drug delivery systems (DDSs) such as nanoparticles and hydrogels usually cannot provide effective treatment due to rapid clearance and degradation or low bioavailability. In this study, a ROS-responsive nanofiber membrane named PLA/PEGDA-EDT@rGO-Fucoxanthin (PPGF) is fabricated by electrospinning, wherein PEGDA-EDT served as the ROS-responsive motif, reduced graphene oxide (rGO) as the drug carrier and fucoxanthin (Fx) as the antioxidative and anti-inflammatory agent. The results demonstrated that the PPGF nanofiber membrane exhibited sustained and long-term Fx release behavior (at least 66 days) in response to hydrogen peroxide (H_2_O_2_) in vitro. With low cytotoxicity and smart ROS responsiveness, PPGF showed excellent anti-inflammatory and antioxidative effects on IL-1β-induced chondrocytes by potent ROS scavenging potential and upregulation of antioxidative enzymes. It also demonstrated the attenuation of OA progression with the reduced Osteoarthritis Research Society International (OARSI) score by 93.17% in 8 weeks. The smart ROS-responsive, biodegradable and biocompatible nanofiber membranes possess great potential for OA therapy under arthroscopy.

## Introduction

Osteoarthritis (OA) which is distinguished by synovial inflammation, cartilaginous degradation, and damage to the subchondral bone, is a global common chronic and progressive joint disease and may lead to pain and disorders^1^. During the OA progression, reactive oxygen species (ROS) are considered the main mechanism of OA, which cause oxidative stress to disrupt cartilage homeostasis and induce cell apoptosis and deoxyribonucleic acid (DNA) damage in chondrocytes. ROS may activate pro-inflammatory cytokines including matrix metalloproteinases (MMPs) and interleukin-1 (IL-1β), to further increase ROS generation in turn^2–4^, contributing to cartilage degradation and OA pathogenesis^5^.

Intra-articular (IA) injection of ROS-responsive drug delivery systems (DDSs) that are controllable to release therapeutic drugs upon the reaction with ROS and other free radicals is considered a promising therapeutic strategy for OA. ROS-responsive nanoplatforms like PEGylated and phenylboronic acid-modified levodopa nanoparticles, poly (lactic-co-glycolic acid)-based nanoparticles, etc. have been applied to deliver the therapeutic drug into the OA joints. They can smartly respond to excessive ROS and trigger sustained drug release according to the severity of OA, exerting prominent therapeutic efficacy^6–8^. However, the dense extracellular matrix (ECM) of cartilage prevents the penetration of nanoparticles due to the unspecific targeting to the cartilage surface and fast clearance, which requires repetitive high-dose administration in the joint cavity and may have the risk of postoperative infection^9–11^. Electrospun nanofiber membranes have been developed for DDSs due to their high drug encapsulation and loading capacity, ability to modulate the release of biomolecules, and ease of processing^12^. The extremely large surface area and porosity of electrospun nanofiber membranes enable the sustained delivery and sensitivity to specific signals^13^. They are suitable for topical drug delivery in a minimally invasive way such as arthroscopy, which may increase the local drug concentrations in the joint and reduce the frequency of drug administration^14,15^. Thus, ROS-responsive nanofiber membranes may shed light on OA therapy, although it has not been reported yet.

The introduction of ROS-sensitive motifs is important for ROS-responsive DDSs, which enables the disassembly of the system in an oxidative environment through carrier degradation and solubility change^16,17^. There are various ROS-responsive functional moieties applied in DDSs, such as polyproline, peroxalate ester, boronic ester, aminoacrylate, polysaccharide, thioketal, selenium/tellurium, and thioether^18–20^. Poly (ethylene glycol) diacrylate (PEGDA)-1, 2-ethanedithiol (EDT) copolymer (PEGDA-EDT) is one of the ROS-responsive materials, which converts hydrophobic thioether groups into hydrophilic sulfone and sulfoxide groups in responses of ROS. This motif has been applied for ROS-responsive delivery of anti-oxidants to protect the cells from oxidative damage, with the result of reduced oxidative stress and restoration of the extracellular physiological state both in vitro and in vivo, demonstrating its safety and potential for bio-applications^21,22^.

For most fiber membranes as DDSs, long-term and sustained drug release can hardly be attained because they only depend on the diffusion-based release of drugs, which degrade within the range of nearly 35 days. Introducing the nanoparticles such as mesoporous silica or bioactive glass nanospheres as drug carriers into the nanofiber membranes prolonged the release time of the drug up to >60 days^23,24^. However, most nanoparticles have inferior biocompatibility and are unfavorable for cell growth^25^. As one of the graphene-based nanomaterials, reduced graphene oxide (rGO) has recently emerged as a potential drug carries for various diseases due to its favorable biocompatibility and unique tunable physicochemical properties, such as extremely large surface area, modifiable active groups, etc^26,27^. The π–π conjugate system on a hydrophobic flat region of rGO makes it easy to load genes, chemical drugs, and other molecules^28^. GO-based composite nanofibers have been successfully prepared by electrospinning. Gao et al.^29^ prepared biomimetic gelatin-based electrospun composite fibers containing chitosan, hydroxyapatite (HA), and GO to simulate the ECM for bone regeneration. Lu et al.^30^ fabricated rGO/chitosan/poly (vinyl alcohol) nanofiber scaffolds for wound healing and found that rGO is beneficial for cellular attachment and growth.

**Fig. 1:**
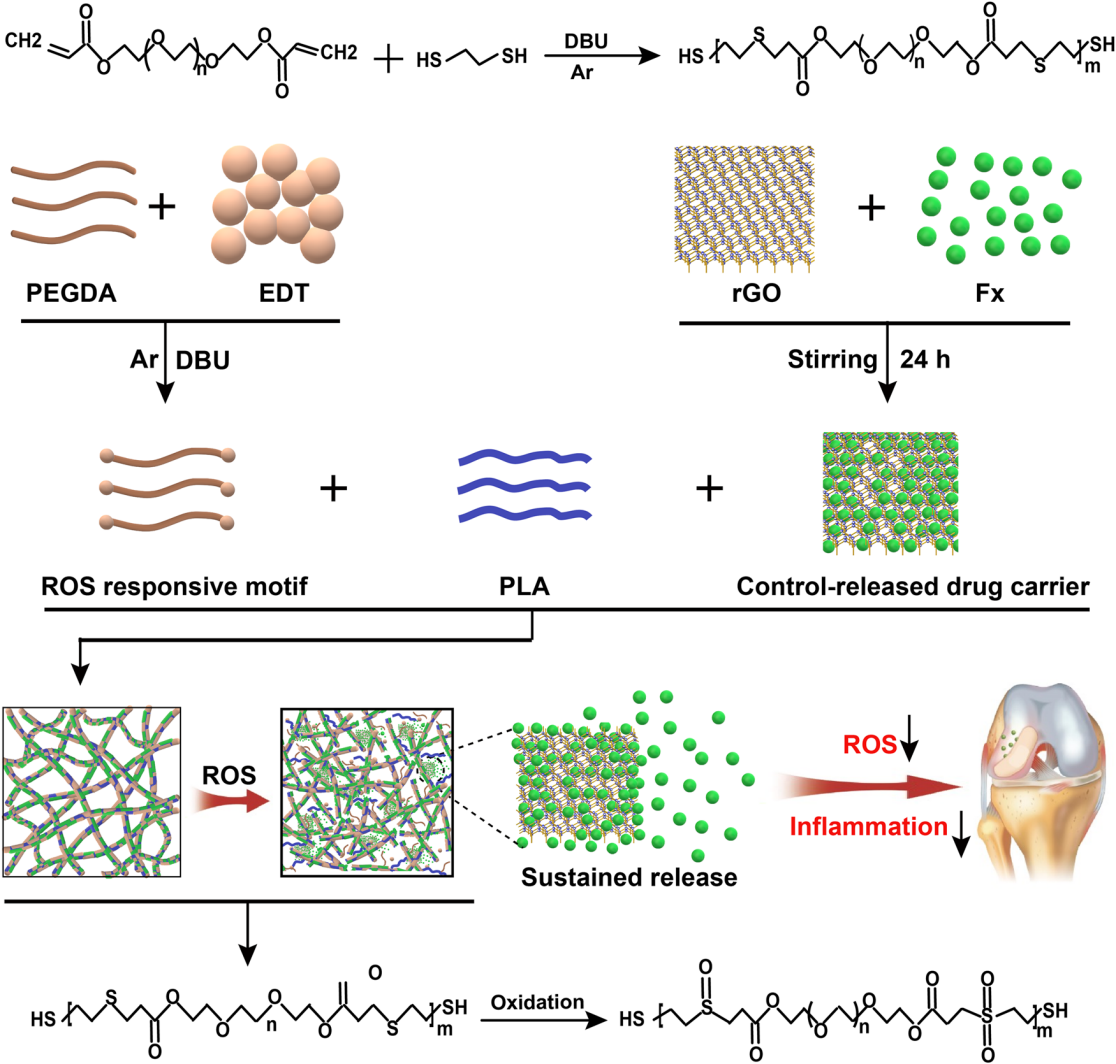
Schematic illustration of the PPGF nanofiber membrane as a ROS-responsive drug delivery system for sustained release of Fx in OA treatment.

In this study, we fabricated an intelligent ROS-responsive poly (lactic acid) (PLA)/PEGDA-EDT@rGO-Fucoxanthin (PPGF) nanofiber membrane as DDSs for OA therapy, wherein PLA served as the backbone, PEGDA-EDT as the ROS-responsive motif, and rGO as a drug carrier, with the intention to achieve smart responsiveness followed by long-term sustained drug release (Fig. 1). fucoxanthin (Fx), the dominant carotenoid in brown seaweed, is a marine drug with potent antioxidative and anti-inflammatory potential, and has been reported to attenuate arthritis^31,32^. This novel PPGF system may potentiate the on-demand release of the drug in response to ROS and greatly increase the retention time of drug, thereby promoting therapeutic efficacy. The smart ROS-responsive DDSs may provide a novel strategy for OA therapy under arthroscopy.

## Results and discussion

### Synthesis and characterization of nanofiber membranes

#### Characterization of synthesized PEGDA-EDT

**Fig. 2:**
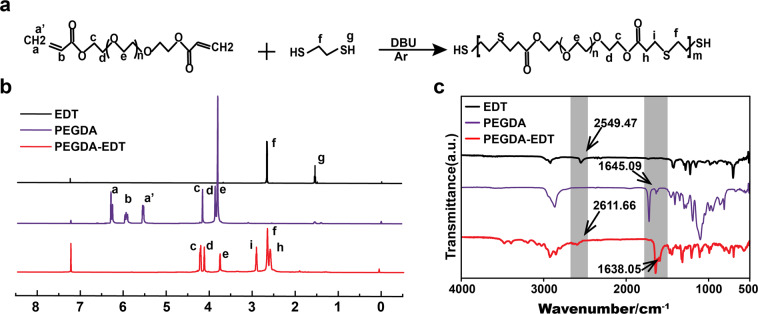
The synthetic route and characterization of PEGDA-EDT. **a** Synthetic route of PEGDA-EDT. **b** ^1^H-NMR and **c** FTIR spectra of the PEGDA-EDT.

PEGDA-EDT copolymer was synthesized using PEGDA and EDT based on the thiol-ene click reaction (Fig. 2a). The structural changes of the synthetic PEGDA-EDT were investigated by Fourier Transform Infrared Spectroscopy (FTIR) and Hydrogen Nuclear Magnetic Resonance (^1^H-NMR). The ^1^H-NMR was performed in CDCl_3_ to characterize the monomers and the synthesized polymer (Fig. 2b). In PEGDA-EDT, the original peak representing sulfhydryl in EDT (g) and vinyl in PEGDT (a, b, a’) disappeared. In the spectrum of PEGDA-EDT, the peaks corresponding to the ester groups in the linkage of hydrophobic segments at *δ* = 2.88~2.78 ppm (i) and that of hydrophilic segments at 2.70~2.60 ppm (h) appeared, suggesting the successful thiol-ene polymerization and formation of the periodical β-thiopropionate group in backbone.

As shown in FTIR spectrum, a new characteristic absorption bands indicating sulfhydryl group (2611.66 cm^−1^) was observed in synthesized PEGDA-EDT, due to the introduction of EDT (2549.47 cm^−1^) (Fig. 2c). As consumed by thiol−ene reaction, the absorption peak of -C=C- in PEGDA-EDT at ~1638.05 cm^−1^ was intensively decreased compared with that in PEGDA (1645.09 cm^−1^). The results confirmed the successful conjunction between PEGDA and EDT.

#### Fabrication and characterization of nanofiber membranes

**Fig. 3:**
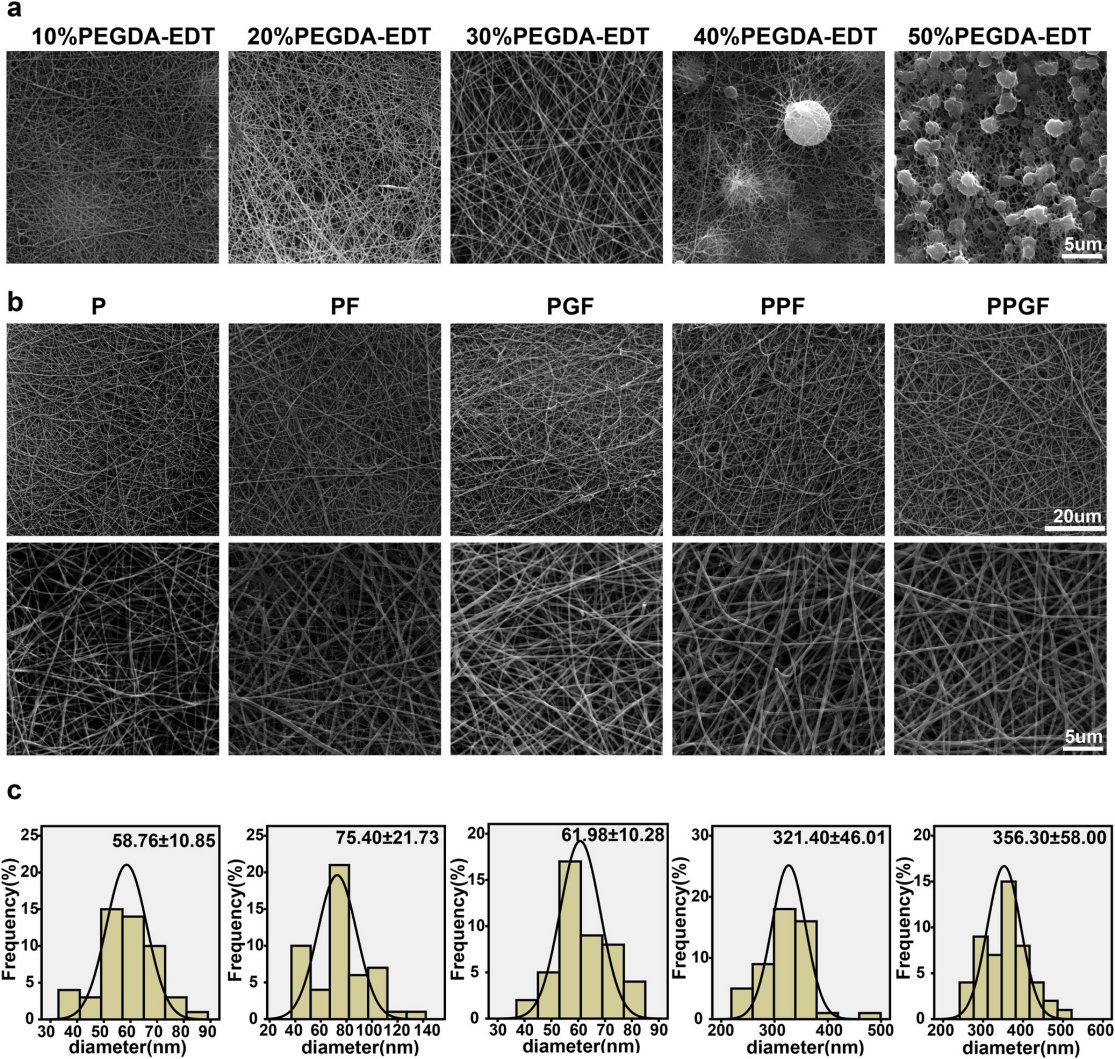
The morphology and diameter of nanofiber membranes. **a** The appearance of nanofiber membranes fabricated by PLA and different concentrations of PEGDA-EDT was observed by SEM (Scale bar, 5 µm). **b** The morphological features and average diameters of P, PF, PGF, PPF, and PPGF nanofiber membranes (Original low magnification: scale bar, 200 μm; original high magnification: scale bar, 5 μm). **c** The diameter distribution of P, PF, PGF, PPF, and PPGF nanofiber membranes was calculated according to **b**.

To determine the concentration of PEGDA-EDT for electrospinning, the morphology of nanofiber membranes fabricated by PLA and different concentrations of PEGDA-EDT were observed by scanning electron microscope (SEM). The diameters were increased gradually with the increase in the concentration of PEGDA-EDT (Fig. 3a). However, fibers were barely produced and beads were deposited when the concentration of PEGDA-EDT was up to 40%, due to the increase of viscosity. Therefore, 30% of PEGDA-EDT was chosen to prepare the PPF and PPGF nanofiber membranes for further experiments.

The optimal concentration of Fx was screened according to the 3-(4,5-Dimethylthiazol-2-yl)-2,5-diphenyltetrazolium bromide (MTT) assay. Cell viability was the highest when treated with Fx at concentrations ranging from 20 to 50 μM, especially at 30 μM (Supplementary Fig. S1). Concentration of Fx above 90 μM exhibited significant cytotoxicity, as evidenced by dramatical decline of cell viability. Assessed by the mass discrepancy, the loading efficiency of Fx in the rGO was 51.85%. It has been reported that graphene has at least a double external surface area favoring the conjunction of drugs, genes, and other molecules^33,34^, which has been widely used in drug delivery applications^35^.

As evidenced by energy-dispersive spectrometry (EDS) analysis, sulfur elements only existed in the PPF and PPGF groups, which contained the PEGDA-EDT (Supplementary Fig. S2). The morphology of fabricated nanofiber membranes including P, PF, PGF, PPF and PPGF was observed by SEM. Fibers in all membranes were smooth and uniformly distributed and no beads were observed (Fig. 3b). With the introduction of substances increased, the diameter of P, PF, PGF, PPF, PPGF nanofiber membranes was increased gradually (Fig. 3c).

### ROS-responsive release profiles of nanofiber membranes

**Fig. 4:**
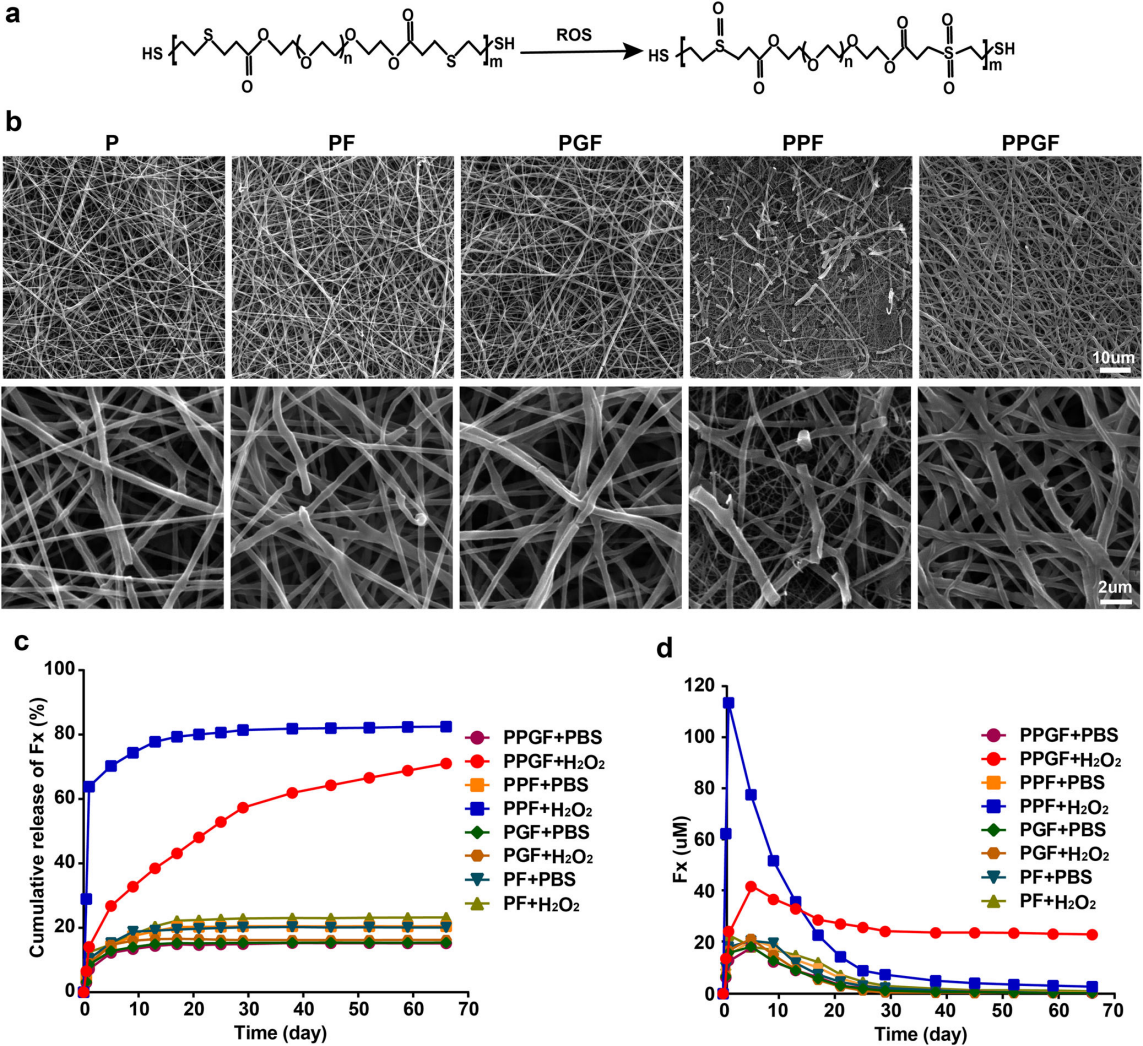
Drug release and ROS responsiveness of nanofiber membranes. **a** The chemical structure of PEGDA-EDT before and after oxidation. **b** The appearance of fibers in different nanofiber membranes after soaking in H_2_O_2_ was observed by SEM (Original low magnification: scale bar, 10 μm; original high magnification: scale bar, 2 μm). **c** In vitro cumulative drug release profile of PF, PGF, PPF, and PPGF in PBS with or without H_2_O_2_. **d** The real-time concentration of drug released from PF, PGF, PPF, or PPGF nanofiber membranes in PBS with or without H_2_O_2_.

In response of ROS, the hydrophobic thioether groups of PEGDA-EDT would be oxidized quickly into sulfone or sulfoxide groups^21^. Figure 4a exhibited the chemical change of PEGDA-EDT copolymer reacted with ROS. The morphology of nanofiber membranes after soaking in PBS with 100 μM of hydrogen peroxide (H_2_O_2_) for 7 days was shown in Fig. 4b, in which, H_2_O_2_ is the most stable and abundant type of ROS to cause oxidative stress^36^. For P, PF, and PGF which were fabricated without PEGDA-EDT, the structure of fibers was well preserved with only a few fractures. In comparison, fibers in PPF and PPGF with ROS-responsive motifs showed obvious destruction. Especially in PPF, some of the long thread-shaped fibers were disrupted into small debris. For both PPF and PPGF, PEGDA-EDT tends to be hydrated after reacting with ROS, facilitating the degradation of fibers and the diffusion of drug^22^. However, PPGF showed less disintegrated fibers than PPF, possibly due to the protection of rGO against fast degradation.

In the absence of H_2_O_2_, the cumulative release rates of Fx in all nanofiber membranes were low, within the range of 15.19~23.24% (Fig. 4c). For PF and PGF, the cumulative release rate changed little in the presence of H_2_O_2_. However, the cumulative drug release rates of PPF and PPGF with ROS-responsive PEGDA-EDT increased sharply to above 70%, demonstrating their responsiveness in ROS environment. For PPF, a burst release of Fx occurred within 1 d with the release rate up to almost 63.84%. And the corresponding concentration of Fx was 112.91 μM which was far beyond the efficiency of Fx (30~50 μM) (Fig. 4d). After the oxidative reaction, the hydrophilic groups of nanofiber membranes can absorb the water, resulting in the swelling of hydrogels which facilitates the high-content and fast release of drug^37,38^. In contrast, sustained release of Fx was observed in PPGF with rGO as a drug carrier, which showed a gradual release of Fx from day 1 to 66 days. Fx release from rGO in vitro only lasted 25 days (Supplementary Fig. 3). The introduction of rGO into the hydrogel can decrease the water uptake capacity, leading to low swelling and sustained drug release^39^. Thus, the cooperation of PEGDA-EDT and rGO endowed PPGF nanofiber membrane with ROS responsiveness and long-term release of the drug.

### Cell viability

**Fig. 5:**
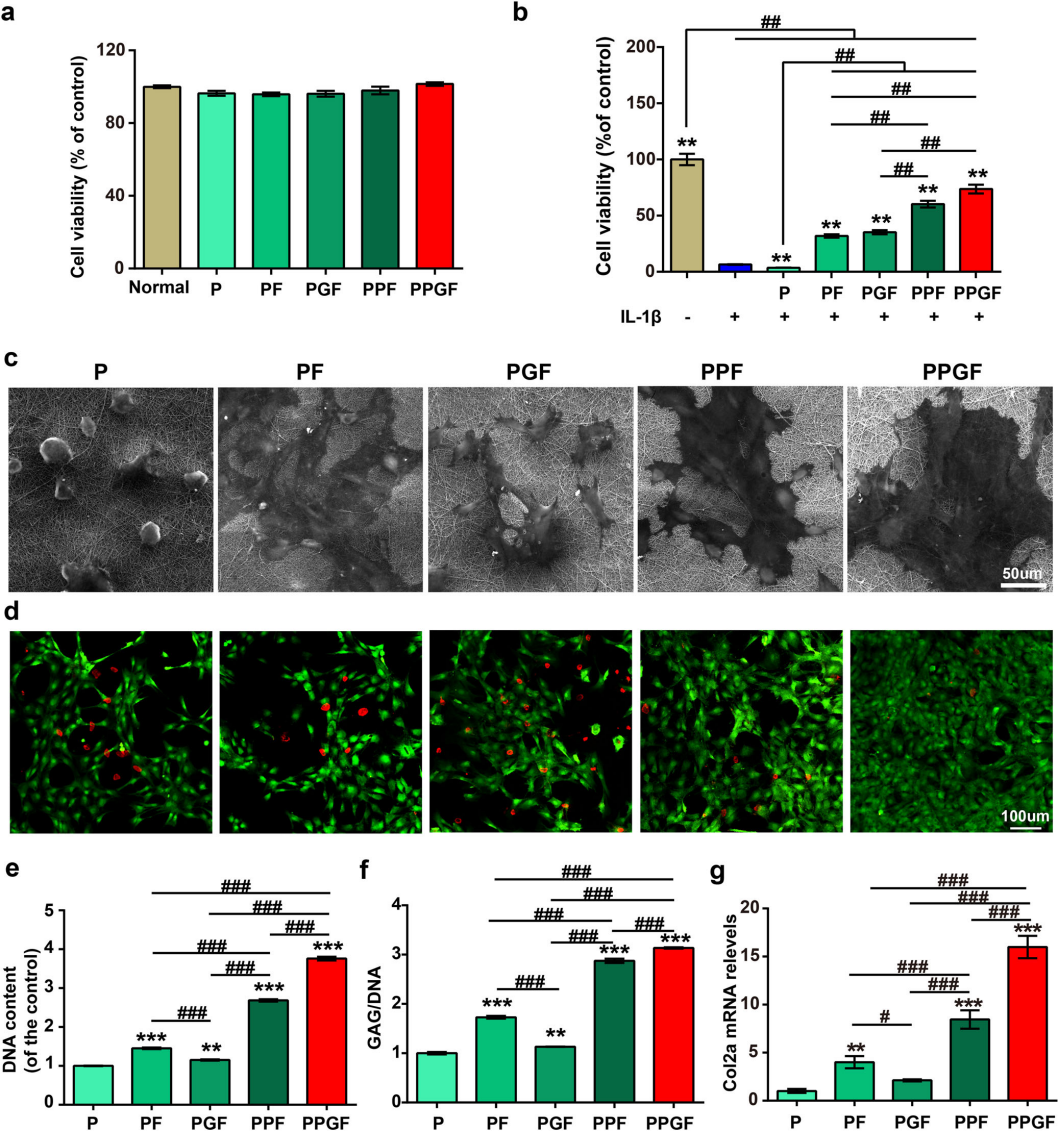
Chondroprotective effects of nanofiber membranes on IL-1β stimulated chondrocytes. **a**, **b** Cell viability of chondrocytes cultured on different nanofiber membranes in the stimulation with or without IL-1β (**^, ##^ means *p* < 0.001, * is the statistical difference compared with IL-1β group and ^#^ is the statistical difference between the pairwise comparison). **c** Cell attachment of chondrocytes cultured on different nanofiber membranes (Scale bars, 50 µm). **d** Live/dead assay of chondrocytes cultured on different nanofiber membranes after induced by IL-1β (Scale bars, 100 µm). **e** DNA and **f** GAG contents of chondrocytes cultured on different nanofiber membranes after being induced by IL-1β. **g** Relative mRNA expression levels of Col2a1 of chondrocytes cultured on different nanofibrous membranes after induced by IL-1β. The values were presented as the mean ± SD (*n* = 3; statistics: one-way ANOVA;*^, #^ means *p* < 0.05, **^, ##^ means *p* < 0.01, ***^, ###^ means *p* < 0.001, * is the statistical difference between the *P* and Fx-loaded groups, and ^#^ is the statistical difference between the pairwise comparison among the Fx-loaded groups).

Cytotoxicity of P, PF, PGF, PPF, and PPGF nanofiber membranes on chondrocytes induced with or without IL-1β was detected by viability assay kit (CCK-8). The viability of chondrocytes without IL-1β stimulation was unaffected by nanofiber membranes, as compared with normal control, indicating the favorable cytocompatibility of the fibers (Fig. 5a). PLA is biodegradable and biocompatible polyester and the PLA-based formulations have been approved by Food and Drug Administration (FDA) for multiple applications^40^. Fx was reported with low cytotoxicity and has been widely applied in biomedical applications^41^. And rGO is widely applied for tissue engineering, biosensing, drug delivery, and so on for its biocompatible property^42^. Thus, compounded polymers have little cytotoxicity.

After stimulation by IL-1β, one of the most classical pro-inflammatory cytokines that contribute to the progression of OA^43^, cell viability was dramatically dropped (Fig. 5b). This suggested the oxidative stress injury induced by IL-1β. There was little difference between IL-1β and *P* groups, suggesting that *P* has little effect on the recovery of chondrocytes injured by IL-1β. However, the other four nanofiber membranes rescued the IL-1β reduced cell viability in the order of PPGF > PPF > PF > PGF. Particularly, the cell viability was only reduced 32.8% and 12.56% in PPF and PPGF groups, much higher than that in PF and PGF groups. Of all the groups, PPGF group showed the highest cell viability. The results indicated that PPGF effectively prevents the reduction of cell viability induced by IL-1β via the synergic effect of ROS-responsive motif and drug carrier rGO.

### Chondroprotective effect of PPGF

Cells were all well attached on the surface of the nanofiber membranes (Fig. 5c). The live/dead cells in different nanofiber membranes were detected by live/dead staining. Compared to group P, dead cells (red) were decreased and live cells (green) were increased in PF, PGF, PPF, and PPGF groups (Fig. 5d). Slight increase of live cells was shown in PF and PGF groups. But in ROS-responsive nanofiber membranes PPF and PPGF, especially the PPGF, few dead cells and a large number of live cells were presented.

Cell proliferation of IL-1β-induced chondrocytes cultured on different nanofiber membranes was analyzed by DNA content. In accordance with the results of the cell viability assay and live/dead staining, cell proliferation was enhanced by nanofiber membranes added with Fx (Fig. 5e). The DNA content was increased in the order of PPGF > PPF > PF > PGF. Particularly, DNA content was the highest in PPGF, demonstrating that PPGF greatly supported cell proliferation after stimulation of IL-1β.

As the main component of cartilage ECM, glycosaminoglycans (GAG) were analyzed by 1, 9-dimethylmethylene blue (DMMB) assay and then normalized by the DNA content. As shown in Fig. 5f, GAG loss induced by IL-1β was slightly recovered by PF and PGF. But the GAG synthesis was remarkably enhanced in the ROS-responsive PPF and PPGF nanofiber membrane, compared with the control (P). In particular, the PPGF nanofiber membrane showed the highest production of GAG content, which is almost two times higher than P.

The gene expression profile of collagen type II alpha 1 chain (*Col2a1*), specific for articular cartilage, confirmed the results of the GAG content. The trend of gene expression increased along the PPGF > PPF > PF > PGF > P (Fig. 5g). In the comparison with P, level of *Col2a1* was increased slightly in the PF and PGF groups. However, it was increased by 7.33 and 14.78-folds in the PPF and PPGF group. The results indicated that ROS-responsive PPF and PPGF, especially PPGF, effectively restored cartilage matrix loss by IL-1β.

### PPGF inhibited inflammatory induced by Il-1β

**Fig. 6:**
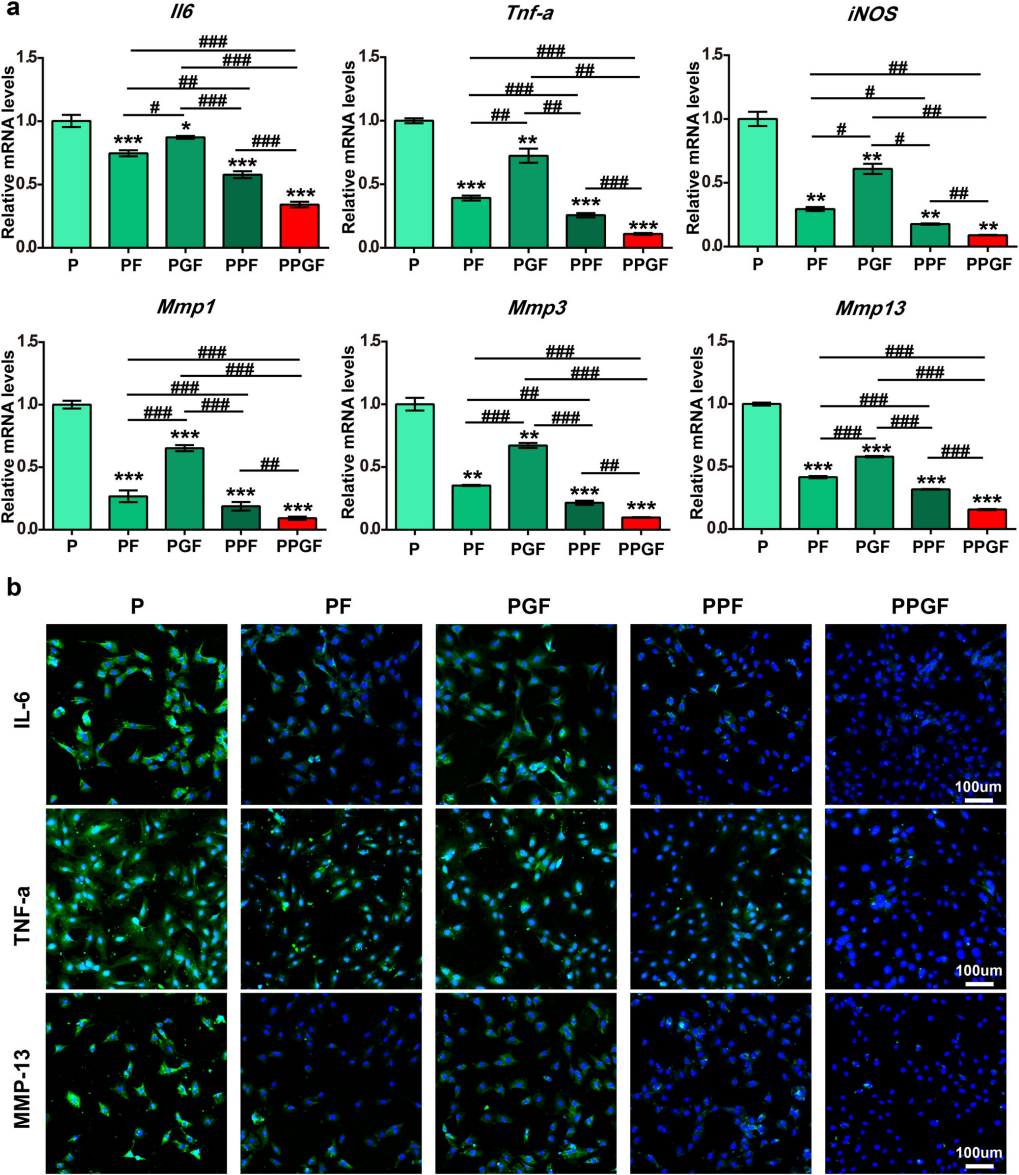
Inhibitive effect of nanofiber membranes on anti-inflammation in Il-1β induced chondrocytes. **a** Relative mRNA expression levels of *Il6, Tnf-a, iNos, Mmp1, Mmp3, and Mmp13* in IL-1β induced chondrocytes cultured on P, PGF, PF, PPF, or PPGF nanofiber membranes. **b** Protein expression of MMP13, TNF-a, and IL6 in IL-1β induced chondrocytes cultured on P, PGF, PF, PPF, or PPGF nanofiber membranes (Scale bars, 100 µm). The values were presented as mean ± SD (*n* = 3; statistics: one-way ANOVA; ^#^ means *p* < 0.01, **^, ##^ means *p* < 0.01, ***^, ###^ means *p* < 0.001, * is the statistical difference compare with P group and ^#^ is the statistical difference between the pairwise comparison).

The anti-inflammatory effect of the nanofiber membranes was evaluated by real-time quantitative polymerase chain reaction (qRT-PCR). The expression levels of inflammatory markers including interleukin 6 (*Il6*), tumor necrosis factor (*Tnf-α*), nitric oxide synthase 2 (*iNos*), and catabolic biomarkers including matrix metalloproteinases 1 (*Mmp1*), matrix metalloproteinases 3 (*Mmp3*), matrix metalloproteinases 13 (*Mmp13*) were significantly inhibited by the nanofiber membranes (PF, PGF, PPF, and PPGF) compared to P (*p* < 0.05) (Fig. 6a). The levels of these markers showed the trend of expression along PGF > PF > PPF > PPGF. PPGF and PPF exhibited evident down-regulation of the expression of the inflammatory markers which were elevated by IL-1β. Particularly, PPGF showed the highest decrease in the expression of these inflammation-related cytokines in all the groups. There was a 65.86%, 89.65%, and 91.03% decline for *Il6*, *Tnf-α*, and *iNo* and a 90.88%, 90.34%, and 84.35% decline for *Mmp1*, *Mmp3,* and *Mmp13* respectively, compared with P.

The protein expression of inflammatory markers, including MMP13, TNF-α, and IL6, in chondrocytes cultured on different nanofiber membranes was examined by immunofluorescence staining. It showed strong positive staining (green fluorescence) of MMP13, TNF-α and IL6 in P and negative staining in Fx-loaded nanofiber membranes (PF, PGF, PPF, and PPGF) which was in accordance with the results of qRT-PCR (Fig. 6b). Studies have demonstrated Fx can suppress the upregulation of pro-inflammatory factors including COX-2, IL-1β, TNF-α, and iNOS mediated by LPS-induced macrophages by inhibiting the phosphorylation of Akt, MAPKs, and IκB-α^44,45^. Fx was also reported to the potential in the treatment of arthritis by reducing the generation of NO and osteoclast differentiation^32,46^. Among the five nanofiber membranes, PPGF showed the lowest expression of inflammatory cytokines, indicating the potent effect on anti-inflammation.

### PPGF suppressed oxidation stress induced by IL-1β

**Fig. 7:**
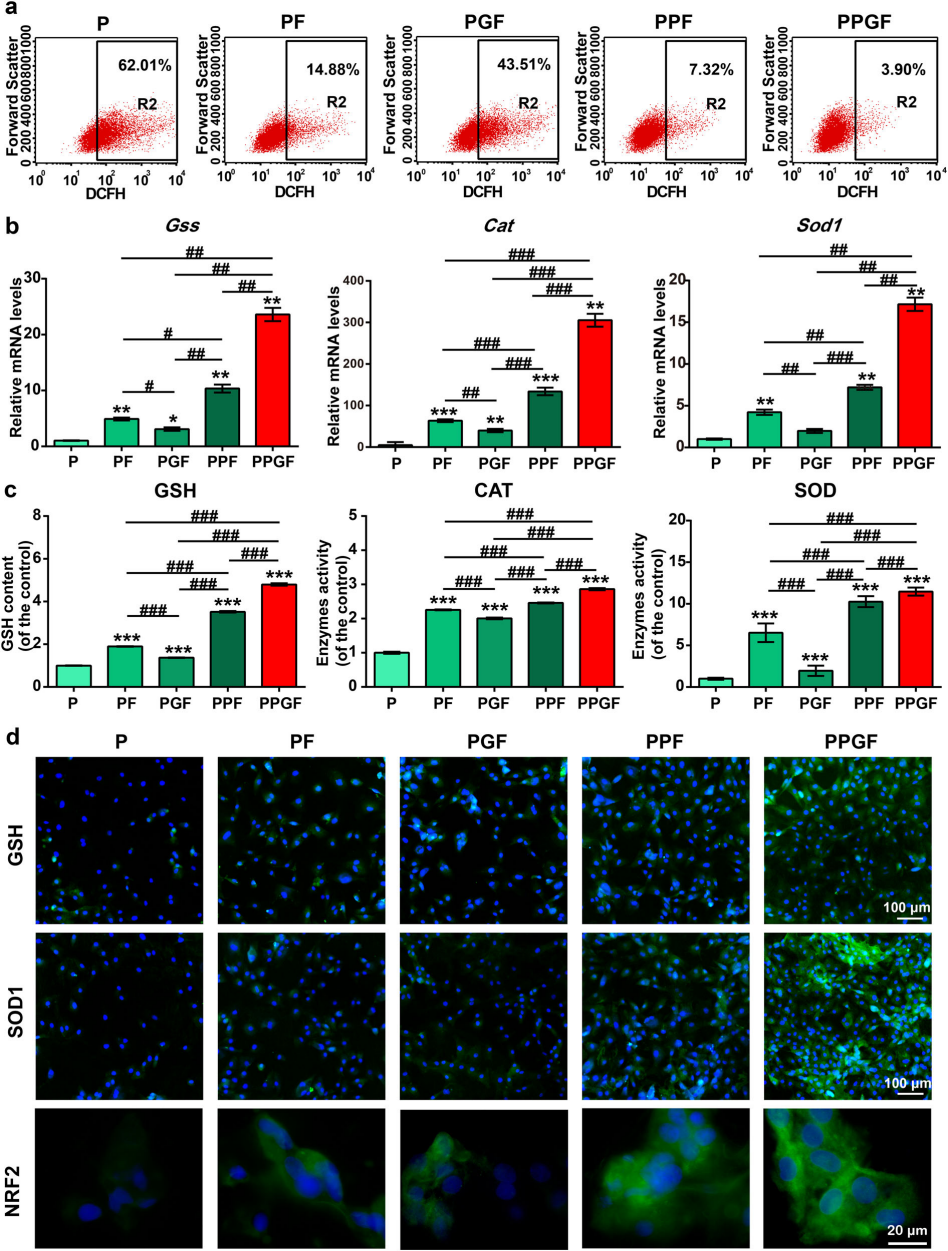
Suppressive effect of nanofiber membranes on oxidative stress in Il-1β induced chondrocytes. **a** ROS content in IL-1β-induced chondrocytes administrated with nanofiber membranes was evaluated by flow cytometry. **b** The expression of antioxidative related genes, including *Cat, Gss,* and *Sod1*, in Il-1β induced chondrocytes treated with nanofiber membranes. **c** Expression of antioxidative factors, including GSH, CAT, and SOD, in Il-1β-induced chondrocytes treated with nanofiber membranes, was detected by ELISA. **d** Expression of GSH, SOD1, and NRF2 in Il-1β-induced chondrocytes treated with nanofiber membranes was detected by immunofluorescence staining. (Scale bars, 100 µm and 20 µm). The values were presented as mean ± SD (*n* = 3; statistics: one-way ANOVA; *^, #^ means *p* < 0.05, **^, ##^ means *p* < 0.01, ***^, ###^ means *p* < 0.001, * is the statistical difference compare with *P* and ^#^ is the statistical difference between the pairwise comparison).

Flow cytometry was used to determine the levels of intracellular ROS in chondrocytes induced by Il-1β. The ROS was raised by IL-1β to 62.01%, but inhibited by drug-loaded nanofiber membranes including PF, PGF, PPF, and PPGF in the order of PPGF > PPF > PF > PGF (Fig. 7a). In all the groups, PPF and PPGF showed the most prominent decline of ROS. Especially, PPGF decreased the ROS levels the most, with only 3.90% of ROS left.

The activity of antioxidative factors in IL-1β-induced chondrocytes cultured on different nanofiber membranes was detected by qRT-PCR, enzyme-linked immune sorbent assay (ELISA), and immunofluorescence staining. As shown in Fig. 7b, gene expression levels of glutathione synthetase (*Gss*), catalase (*Cat*) and superoxide dismutase 1 (*Sod1*) showed the trend of PPGF > PPF > PF > PGF. PPF and PPGF evidently upregulated the expression levels of the *Gss*, *Cat,* and *Sod1*. Particularly, PPGF promoted the expression of antioxidative factors the most prominently. Protein expression of glutathione (GSH), CAT, SOD1, and nuclear factor erythroid-2-related factor 2 (NRF2) by ELISA and immunofluorescence staining also confirmed the results of the PCR findings, demonstrating the potent antioxidative effect of PPGF (Fig. 7c, d). NRF2 is a transcription factor that acts as a core role in the regulation of antioxidant^47^. Once activated, it will drive downstream pathways to produce a variety of antioxidative enzymes such as the GSH, CAT, and SOD. SOD catalyzes the conversion of O^2^· to less detrimental H_2_O_2_ which can be further reduced by enzymes like CAT^48^. GSH is a co-factor for glutathione peroxidase activity and a major cellular ROS scavenger^49^. Excessive production of ROS causes lipid peroxidation in chondrocyte^50^ and leads to cell apoptosis which was related to GSH-depleted^51^. CAT can catalyze the H_2_O_2_ reduction into the water which acts a compensatory and protective role in reducing the progression of OA^52^. The results demonstrated the potent antioxidant role of PPGF by upregulation of crucial antioxidative factors.

### The retaining profile of Fx in the joints

**Fig. 8:**
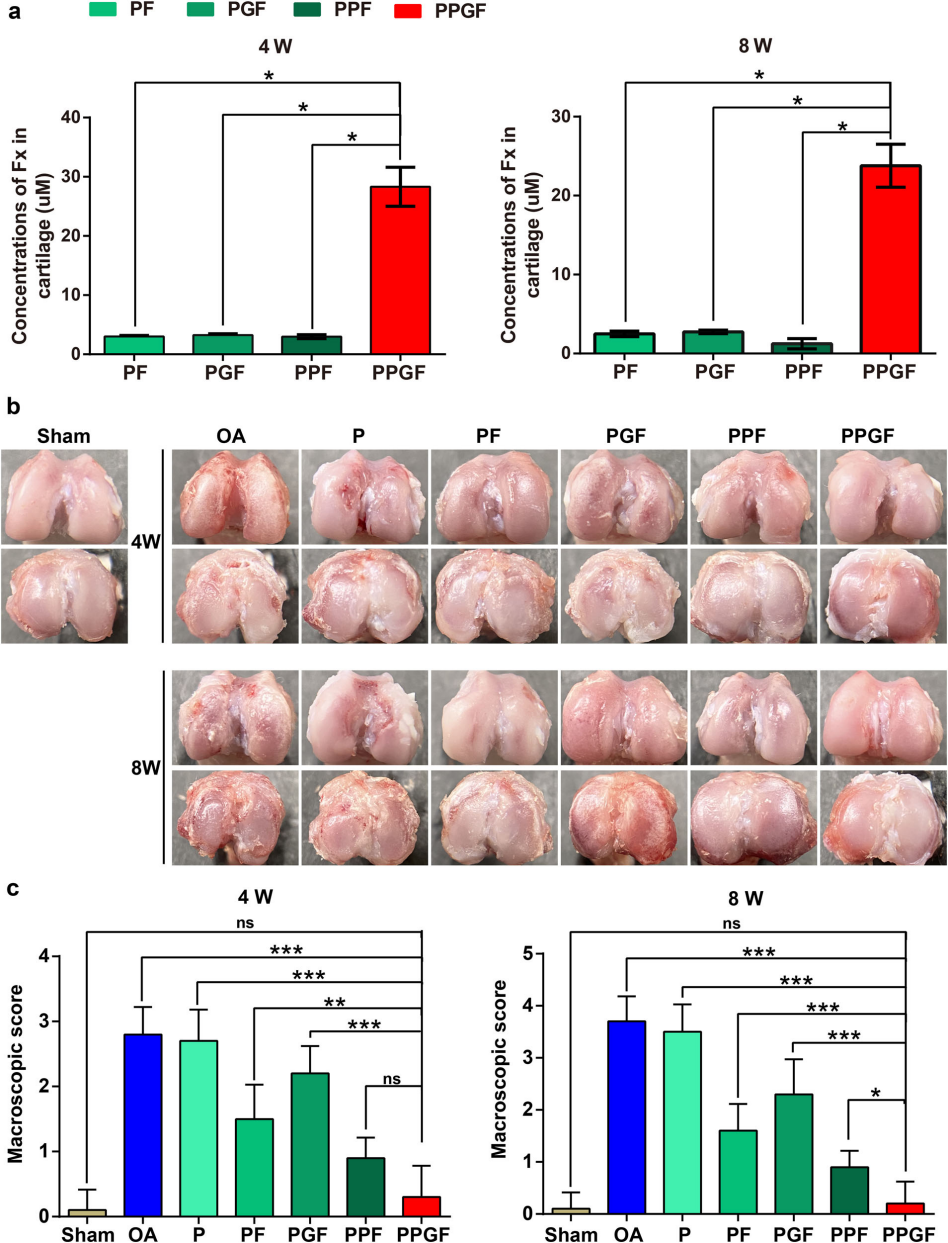
Macroscopic evaluation of articular treated by nanofiber membranes. **a** The concentration of Fx in the cartilage after implantation of the nanofiber membranes for 4 and 8 weeks. **b** Macroscopic appearance and **c** score of articular from the distal femur and tibial plateau after treatment by nanofiber membranes for 4 and 8 weeks. The values were presented as mean ± SD (*n* = 5; statistics: one-way ANOVA; ns means *p* > 0.05, * means *p* < 0.05, ** means *p* < 0.01, *** means *p* < 0.001).

The retention of Fx in cartilage was analyzed by high-performance liquid chromatography (HPLC). The concentration of Fx in the cartilage was decreased with the treatment time (Fig. 8a). Intra-articular implantation of PF, PGF, or PPF for 4 and 8 weeks exhibited similar concentrations of Fx. (*p* > 0.05). However, Intra-articular implantation of PPGF exhibited a higher level of Fx in cartilage compared to the PF, PGF, or PPF groups. Indicate the long-term drug release profile of PPGF.

### PPGF attenuated OA progression in rats

The macroscopic observation and score of the cartilages from the distal femur and tibial plateau were implemented to access the therapeutic efficiency of nanofiber membranes on OA. Joint cartilages with rough and erosive surface and synovium hyperplasia, which are OA characteristics, were observed in OA groups after 4 weeks (Fig. 8b). In the treatment of P, the cartilage destruction and the degree of degradation were not alleviated, as compared with OA group. The recovery of cartilage lessons was observed in PF, PGF, PPF, and PPGF groups, with the score of P > PGF > PF > PPF > PPGF (Fig. 8c). Particularly, PPGF restored the cartilage damage the most markedly with the smooth and integrated surface, which is almost close to normal cartilage. At week 8, cartilage defect and osteophyte formation were present in OA groups, indicating the deterioration of cartilage destruction with time. The results in 8-week administration by nanofiber membranes were in agreement with that in 4-week treatment. PPGF displayed the most prominent therapeutic effect on OA in all the groups, which decrease the macroscopic scores to 89.29% and 94.59% at 4 and 8 weeks, respectively.

**Fig. 9:**
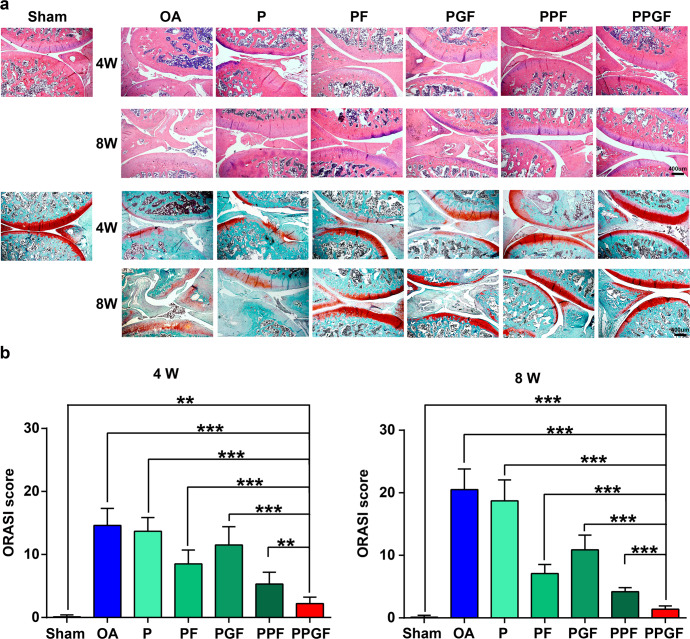
Histological analyses of articular in the treatment of nanofiber membranes. **a** H&E and **b** safranin O/fast green staining of the cartilage after treatment by nanofiber membranes for 4 and 8 weeks. **c** OARSI score of the articular cartilage based on histological staining. The values were presented as mean ± SD (*n* = 5; statistics: one-way ANOVA; ** means *p* < 0.01, *** means *p* < 0.001).

The therapeutic efficiency of nanofiber membranes on OA was further analyzed by the histological examination (Fig. 9). The results were consistent with the macroscopic observation (Fig. 8b, c). As shown by the hematoxylin–eosin (H&E) staining, in the administration of anterior cruciate ligament transaction (ACLT) lead to inflammatory hyperplasia, fibrillation, fissure, vertical cracks, deformation of cartilage, and loss of proteoglycans (Fig. 9a), which were deteriorated over time. In comparison, morphological improvements, such as tidemark integrity, columnar structure of chondrocytes, and smoothness of cartilage surface, were prominently ameliorated by PF, PGF, PPF, or PPGF nanofiber membranes. Positive staining (red staining) indicating the secretion of GAG which is specific for articular cartilage was also shown in Fx-loaded membranes. Particularly, PPGF exhibited almost no cartilage erosion or synovial hyperplasia with strong expression of proteoglycans by safranin O. The results of OARSI score also confirmed that nanofiber membranes loaded with Fx relieved the progress of OA, with the trend of PGF > PF > PPF > PPGF (Fig. 9b). In particular, PPGF showed the most evident therapeutic effect on the alleviation of the severity of OA, which decreased the OARSI score to 84.93% and 93.17% at 4 and 8 weeks, respectively. In our previous work, we synthesized the lignin and poly (ε-caprolactone) (PCL) nanofiber membranes OA therapy by arthroscopic intervention based on the intrinsic antioxidative ability of lignin^9^. Although effective in the delay of OA progression in the early stage, the PCL/lignin can hardly prevent cartilage degradation in the long term. In this study, a combination of ROS responsiveness and long-term drug release may be preferable for membranes in OA treatment. The functional change of the joint was also investigated by giat analysis. As shown in Supplementary Fig. 4, the footprint area and intensity of the rats in PPGF group were significantly higher that OA group (*p* < 0.05).

## Conclusions

In summary, we fabricated a PPGF nanofiber membrane by introducing PEGDA-EDT as ROS-responsive motif, rGO as a drug carrier, and Fx as antioxidative and anti-inflammatory agent into the PLA to achieve ROS-responsive and long-term drug release for OA therapy. The novel nanofiber membrane DDS could “turn on” in response to excessive ROS and prolong the residence time of the Fx in the joint cavity, facilitating the effective concentration of the drug to improve the therapeutic efficiency of OA. Therefore, this system is an ideal candidate as a drug sustained release carrier for OA therapy.

## Experimental sections

### Fabrication and characterization of the nanofiber membranes

#### Synthesis and characterization of PEGDA-EDT

The ROS-responsive motif PEGDA-EDT was fabricated based on the thiol-ene click reaction^21^. Firstly, 2.8 mmol (1.68 g) of PEGDA (*M*_n_ = 600 g/mol, Aladdin, Shanghai, China), 2.94 mmol (0.277 g) of EDT (Macklin, Shanghai, China) and 0.28 mmol (0.43 g) of DBU (Macklin, Shanghai, China) were mixed in a system containing 20 mL of chloroform and then stirred by a magnetic stirring (Changcheng, Zhengzhou, China) for 24 h at room temperature under Ar gas protection. Subsequently, the synthesized polymer was precipitated by ice-cooling ethyl ether and refrigerated at −80 °C for 1 h. Then it was separated and mixed with glacial acetic acid for precipitation again. Finally, purified products were dried at Vacuum Freeze Drying Plant (Foring, Beijing, China) for 72 h and then stored at −80 °C. The reactants and synthesized polymer were characterized by FTIR (Shimadzu, Japan) and Hydrogen Nuclear Magnetic Resonance (^1^H-NMR) Spectroscopy (Bruker, Germany).

#### Concentration screening of PEGDA-EDT for preparation of nanofiber membranes

PEGDA-EDT was dissolved in hexafluoroisopropanol (HFIP; Macklin, Shanghai, China) to prepare different concentration of mixtures (0, 5%, 10%, 15%, 20%, 25%, 30%, 35%, 40%, 50% (w/v)). Then PLA was added into the PEGDA-EDT solutions for electrospinning at a distance of 15 cm between the collector and needle tip. The condition of electrospinning was under the electric field of 15.5 kV and a flow rate of 0.8 mL/h. The nanofiber membranes were obtained via vacuum drying.

#### Fx loading

For Fx loading, rGO was used as a carrier. In brief, 10 mg of rGO (XFNANO, Nanjing, China) was added to the ethanol (50 mL) for preparation of suspension, and then 20 mg of Fx (ABPHYTO, Chengdu, China) was dropped in and stirred gently for 1 d. The resulting liquid was dialyzed through the dialysis tube (3000 kD; Solarbio, Beijing, China) to get rid of the uncombined substances. After drying, rGO-Fx complex was stored at room temperature for subsequent experiments. The drug loading ratio (DL) was calculated by the following formula where M is the weight of rGO-Fx and m is the weight of rGO. The morphology of rGO loaded with Fx or not was examined through an SEM (TESCAN, Czech).

$${{{\mathrm{DL}}}}(\% ) = ({{{\mathrm{M}}}} - {{{\mathrm{m}}}})/{{{\mathrm{M}}}} \times 100$$

#### Preparation and of characterisation the nanofiber membranes

The optimal concentration of PEGDA-EDT was chosen according to the previous step. To fabricate the pre-electrospun solution, 240 mg of PLA was added to 3 mL of PEGDA-EDT solution and stirred magnetically for 24 h at room temperature. These solutions were thoroughly mixed according to the formula in Table 1 for premix preparation. The mixed solution was electrospun into nanofibers according to the condition in the previous step. The surface morphology of the synthesized nanofiber membranes was observed using the SEM. And the fiber diameter distributions in the SEM images were measured by Image J. The EDS of the nanofibrous membranes was also investigated.

**Table 1:** The formula of electrospun premix.

Nanofiber membranes	P	PF	PGF	PPF	PPGF
PLA	240 mg	240 mg	240 mg	240 mg	240 mg
PEGDA-EDT	-	-	-	900 mg	900 mg
Fx	-	1 mg	-	1 mg	-
rGO-Fx	-	-	2 mg	-	2 mg
HFIP	3 mL	3 mL	3 mL	3 mL	3 mL

### ROS responsiveness and Fx release of nanofiber membranes in vitro

In vitro drug release from PF, PGF, PPF, PPGF nanofiber membranes, and rGO was investigated by soaking in PBS containing H_2_O_2_ (100 μM) or not under gentle stirring at 37°C. The Ultraviolet Spectrophotometer (Shimadzu, Japan) was used to measure the dose of released Fx at various time points (0, 0.5, 1, 5, 9, 13, 17, 21, 25, 29, 38, 45, 52, 59 and 66 d). Cumulative release rate (*Q*) was calculated according to the following formula, in which, C_t_ and C_i_ are the concentration of Fx at *t* times and *i* times, *V*_0_ and *V*_i_ are the initial volumes and the volume at *i* times (*V*_0_ = 4 mL, *V*_i_ = 1 mL), W is the total loaded mass of Fx in the nanofiber membrane. The morphological changes of the post-oxidative (treated with 100 μM of H_2_O_2_ for 7 days) nanofiber membranes were observed and photographed by SEM.

$${{{\mathrm{Q}}}}\left( \% \right) = \left(C_tV_0 + \mathop {\sum }\limits_{i = 1}^{t - 1} C_iV_i\right)/W \times 100$$

### Chondrocytes isolation and culture

Chondrocytes were isolated from Sprague Dawley (SD) rats and cultured by enzymatic digestion^53^. Cartilage was harvested and digested by trypsin for 30 min and then incubated with collagenase type II for 3 h. The digested supernate was centrifuged for cell collection. Chondrocytes in passage 2 were utilized for the following experiments. All experiments involving animals were performed following the Animal Ethics Committee standards of the Guangxi Medical University.

### MTT assay

The optimal concentration of Fx in IL-1β-stimulated chondrocytes was screened by MTT assay. For this purpose, 200 μL of chondrocytes suspension were seeded in each well of 96-well plate at a density of 5 × 10^3^ cells/mL. After 24 h of culture, gradient concentrations of Fx (0–100 μM) were added to the well and co-culturing with IL-1β (10 ng/mL) for 1 d. Subsequently, the culture medium was refreshed and added with 10 μL of MTT (5 mg/mL). After incubation for 4 h, crystals were dissolved with dimethylsulfoxide and the absorbance at 490 nm was measured by a Microplate Reader (Thermo Fisher Scientific, USA) to evaluate the viability of chondrocytes.

### Cell treatments

Nanofiber membranes were placed in the bottom of the 96-well or six-well plates and seeded with chondrocytes. The cells were divided into five groups according to the culture conditions: (1) P (PLA) group: chondrocytes cultured on the P nanofiber membranes; (2) PF (PLA@Fx) group: chondrocytes cultured on the PF nanofiber membranes; (3) PGF (PLA@rGO-Fx) group: chondrocytes cultured on the PGF nanofiber membranes; (4) PPF (PLA/PEGDA-EDT@Fx) group: chondrocytes cultured on the PPF nanofiber membranes; (5) PPGF (PLA/PEGDA-EDT@rGO-Fx) group: chondrocytes cultured on the PPGF nanofiber membranes. All groups were stimulated by 10 ng/mL of IL-1β and cultured for 24 h.

### Cell attachment

The morphology and attachment of the cells on the nanofiber membranes were observed by SEM. Cells were fixed with 2.5% of glutaraldehyde (Macklin, Shanghai, China) for 12 h at 4°C. After washing with PBS three times, samples were further fixed with 1% of osmium tetroxide (Ted PELLA Inc., USA) for 1 h. And then gradient alcohol was used for dehydration. After vacuum drying and spraying with gold, SEM was performed by JEOL (Tokyo, Japan) JSM-6300V.

### Cell viability assay

The cell viability of chondrocytes was analyzed by a cell viability assay kit (CCK-8; KeyGEN bio, Jiangsu, China) according to the manufacturer’s protocols. Each well was added with CCK-8 solution and incubated for 4 h. Then the absorbance at 450 nm was detected by a Microplate Reader.

In addition, the live/dead assay was also implemented to detect the viability of the chondrocytes. The nanofiber membranes were seeded with chondrocytes and stimulated by IL-1β for 24 h, then washed by PBS three times and stained with a live/dead staining kit (Thermo Fisher Scientific, USA) in the dark at 37 °C for 5 min. Imaging was photographed using a Laser Scanning Confocal Microscope (LSCM; Leica, Germany).

### Quantitation of DNA and GAG content

DNA and GAG contents were detected to investigate the proliferative effect of nanofiber membranes on and ECM secretion of chondrocytes. Chondrocytes on the nanofiber membranes were collected and digested with proteinase K (Sigma-Aldrich, USA) at 56 °C for 16 h. The DNA content was measured using Hoechst 33258 (Solarbio, Beijing, China) dye and quantified by the stander curve constructed by calf thymus DNA. Total secretion of GAG was measured using DMMB (Sigma-Aldrich, USA) with chondroitin sulfate as standard for building a standard curve. GAG production was qualified on a standard curve and normalized to the total DNA contents.

### qRT-PCR analysis

Total RNA of chondrocytes culture on different nanofiber membranes after being stimulated by IL-1β for 1 d was harvested through a total RNA extraction kit (Magen, Guangdong, China). The RNA was reverse transcript and qRT-PCR was performed at the condition of 95 °C for 10 min, followed by 95 °C for 15 s and 60 °C for 1 min by a detection System (Roche, Switzerland)^54,55^. The mRNA expression of *Col2a1*, *iNos*, *Tnf-α*, *Il6*, *Mmp1*, *Mmp3*, *Mmp13*, *Cat*, *Gss,* and *Sod1*was analyzed. And the primary sequences of the genes are exhibited in Table 2.

**Table 2:** Primer sequences used in qRT-PCR.

Gene name	Forward primer (5′-3′–)	Reverse primer (5′–3′)
*Col2a1*	TGCTGGAAAACCTGGTGATG	GTAACCTCTGTGACCCTTGAC
*iNos*	GGTGAGGGGACTGGACTTTTAG	TCTCCGTGGGGCTTGTAGTT
*Tnf-α*	GAGTGACAAGCCTGTAGCC	CTCCTGGTATGAGATAGCAA
*Il6*	GGCATGACTCTCACAATGCG	ACAGTGCATCATCGCTGTTC
*Mmp1*	TGGACCTGAATATGGACTTGCT	GCTGGATGGGATTTGGGGAA
*Mmp3*	GGCTGTGTGCTCATCCTACC	TGGAAAGGTACTGAAGCCACC
*Mmp13*	GGACAAAGACTATCCCCGCC	GGCATGACTCTCACAATGCG
*Cat*	AGAGGAAACGCCTGTGTGAG	TAGTCAGGGTGGACGTCAGT
*Gss*	AATGCCGTGGTGCTACTGAT	GCAGCTCGTTCTCTATGGCA
*Sod1*	GTGGCATCAGCCCTAATCCA	CACCAGTGTGCGGCCAATGA
*Gapdh*	TCCAGTATGACTCTACCCACG	CACGACATACTCAGCACCAG

### Immunofluorescence staining

The expression of MMP13, TNF-α, IL6, SOD, GSH, and NRF2 was analyzed by immunofluorescence staining. Cell-scaffold composites were immobilized with 4% of paraformaldehyde. H_2_O_2_ and normal goat serum from a SPlink detection kit (ZSGB-Bio, Beijing, China) were used to block any endogenous peroxidase activity and eliminated nonspecific staining. Then cells were incubated with primary antibodies against MMP13 (BA2204, dilution ratio 1:400, Boster, Wuhan, China), TNF-α (bs-10802R, dilution ratio 1:500, Bioss, Peking, China), IL6 (BA4339, dilution ratio 1:400, Boster, Wuhan, China), SOD1 (bs-1079R, dilution ratio 1:500, Bioss, Peking, China), GSH (bs-11756R, dilution ratio 1:500, Bioss, Peking, China) and NRF2 (66504-1-Ig, dilution ratio 1:200, Proteintech, Wuhan, China) overnight in a cool condition. Subsequently, they were incubated with a secondary antibody (Bioss, Beijing, China) for 1 h. The cell nucleus was stained by 2-(4-Amidinophenyl)-6-indolecarbamidine dihydrochloride (DAPI; Solarbio, Beijing, China). Fluorescence imaging was captured by LSCM. All procedures were conducted in the dark.

### ELISA

Oxidative stress parameters, including GSH, CAT, and SOD, were detected by using ELISA kits (Meimian, Jiangsu, China). Chondrocytes on nanofiber membranes were harvested by trypsinization and then analyzed according to the performance manually. A Microplate Reader was utilized to measure the absorbance at wavelengths of 412 nm (GSH), 520 nm (CAT), and 450 nm (SOD), respectively. The contents were calculated based on the standard curves.

### Intracellular ROS detection

A fluorescent 2, 7-dichlorodihydrofluorescein diacetate kit (DCFH-DA; Beyotime, Shanghai, China) was used to determine the levels of intracellular ROS. After incubation with 10 nmol/L of DCFH-DA protected from light at 37 °C for 0.5 h, cells were washed with PBS and detected by a Flow Cytometer (BD, USA).

### Construction of OA model treatment of nanofiber membranes

Totals of 70 rats (200~250 g, male) were used in this study. Pentobarbital sodium (40 mg/kg) was injected intraperitoneally for anesthetization. 60 rats received ACLT operations to establish OA model, and the other 10 rats were recruited into the control group that underwent sham operations without ACLT. 4 weeks later, OA rats were divided into 6 groups randomly and different membranes (volume = 6 mm × 2 mm × 0.5 mm) were attached to the cartilage surfaces of the knee joint. The animals were grouped as followed: (1) Control group: sham operation group; (2) OA group: treated without any membranes; (3) P group: treated with PLA membranes; (4) PF group: treated with PLA@Fx membranes, (5) PGF group: treated with PLA@rGO-Fx membranes; (6) PPF group: treated with PLA/PEGDA-EDT@Fx membranes; (7) PPGF group: treated with PLA/PEGDA-EDT@rGO-Fx membranes.

### Fx retention in articular cartilage

Nanofiber membranes were respectively implanted into the knee joint of OA mice for 4 and 8 weeks to investigate the retention of Fx in the cartilage. Rats were sacrificed and then the cartilage was collected. And then added with PBS and grinded. Follow by centrifugation at 8265×*g* for 10 min. The supernatants were assayed by HPLC.

### Gait, macroscopic, and histological analysis

Gait analysis was performed at week 4 after the therapy by using the VisuGait analysis system (Shanghai Xinruan Information Technology Co., Ltd., Shanghai, China). After that articular cartilage in every group was collected and observed. The macroscopic score was performed through 3 valuators who were blinded to the groups according to the scales of 0–4^53^. After being fixed by 4% of paraformaldehyde for 7d, total knee joints were demineralized for 1 month using ethylene diamine tetraacetic acid (EDTA)-decalcifying solution (Macklin, Shanghai, China). Subsequently, total joints were embedded in paraffin and sliced into sections. Histological staining was carried out using the modified safranin O-fast green FCF Cartilage Stain Kit (Solarbio, Beijing, China) and H&E kit (JianCheng Biotech, Nanjing, China). OARSI system was used for histological evaluation and scored by 3 independent estimators who were not known the treated groups.

### Statistical analysis

Statistical analysis was performed by using SPSS 21.0. Data were presented as mean ± standard deviation (SD). One-way analysis of variance (ANOVA) was used to analyze the differences between the groups. *p* < 0.05 was considered with statistical significance.

### Reporting summary

Further information on research design is available in the Nature Research Reporting Summary linked to this article.

## Supplementary information

Supplemental Materials

REPORTING SUMMARY

## Data Availability

All data associated with this study are present in the paper or the Supplementary Information. All relevant data are available from the authors.
