# Supplementary material for: ROS-responsive PPGF nanofiber membrane as a drug delivery system for long-term drug release in attenuation of osteoarthritis
Source: NPJ Regen Med. 2022 Nov 3;7:66. doi: 10.1038/s41536-022-00254-3 (PMC9630282; doi:10.1038/s41536-022-00254-3)
Supplement: Supplementary file 1 — Supplemental Materials [file 41536_2022_254_MOESM1_ESM.pdf]

Supplementary information

ROS-responsive PPGF Nanofiber Membrane as a Drug Delivery System for Long-term Drug Release in Attenuation of Osteoarthritis

Jianjun Wu[#1,2], Zainen Qin[#1,2,3], Xianfang Jiang[#4], Depeng Fang[1,2], Zhenhui Lu*[1,2,3], Li Zheng*[1,2,3], Jinmin Zhao*[1,2,5]

Jianjun Wu, Zaineng Qin and Xianfang Jiang contributed equally to this work.

*** Co-Corresponding authors:** Zhenhui Lu (Zhenhuilu1989@163.com), Li Zheng **(**zhengli224@163.com**)** and Jinmin Zhao (zhaojinmin@126.com)

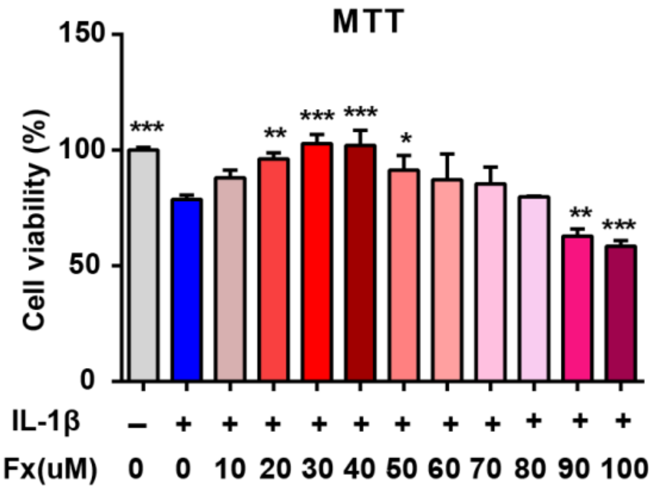

Supplementary Fig. 1 The cytotoxicity of Fx detected by MTT assay.

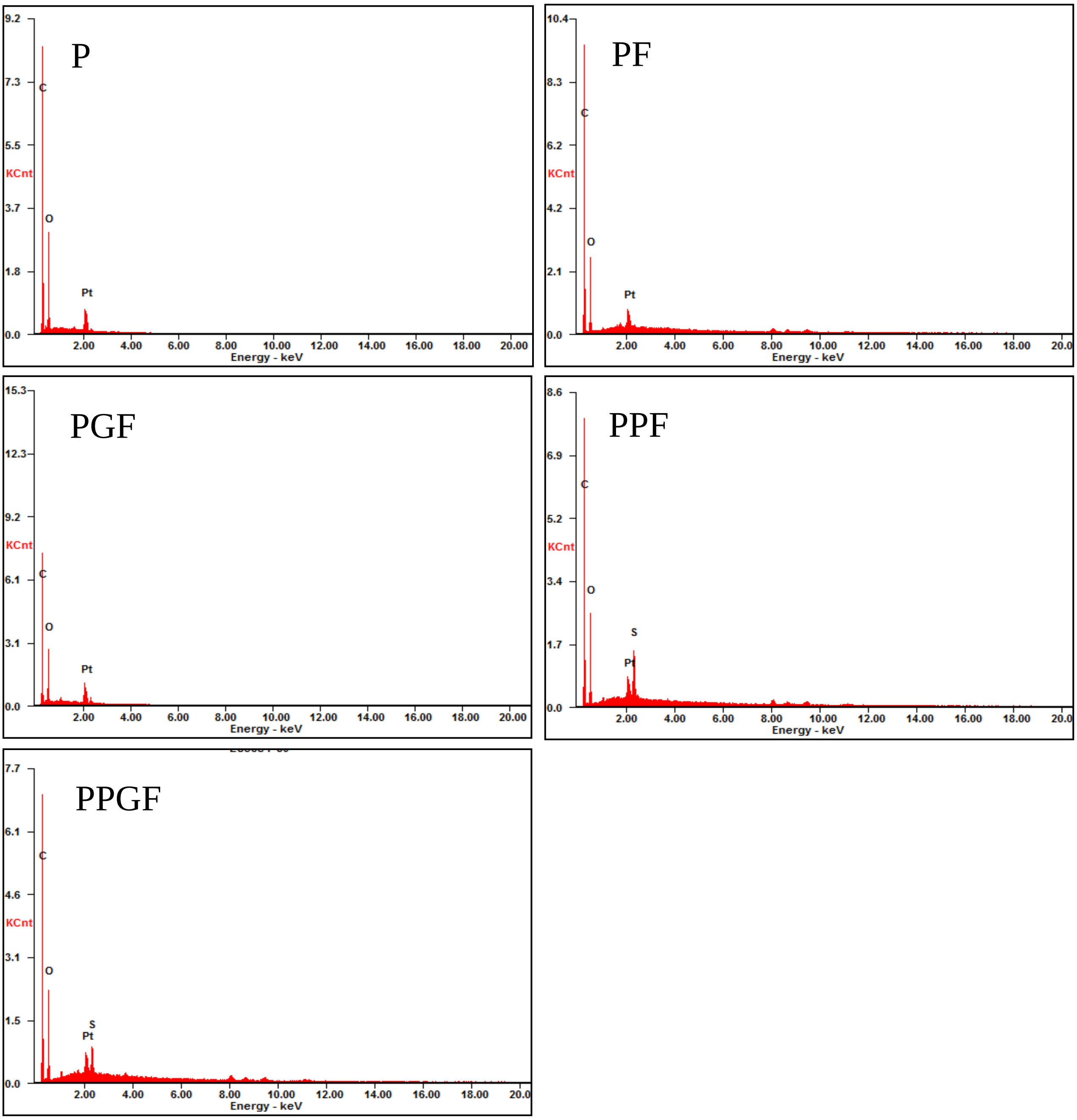

Supplementary Fig. 2 Energy-dispersive spectrometry (EDS) spectrum of nanofiber membranes.

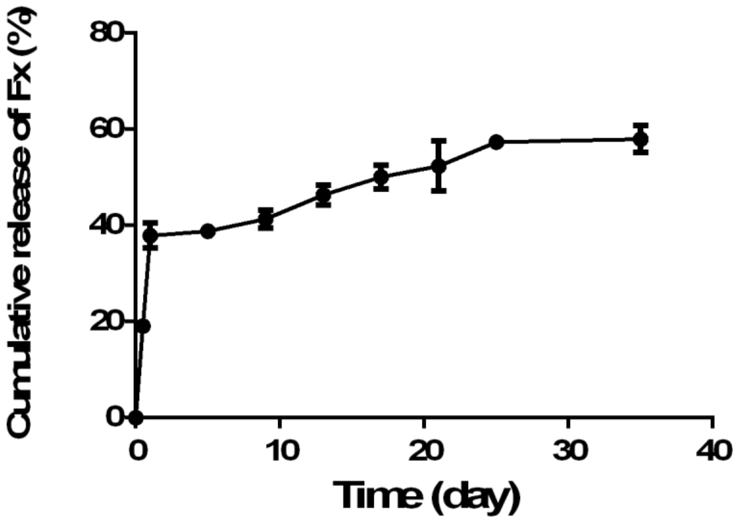

Supplementary Fig. 3 The release profile of Fx from the rGO.

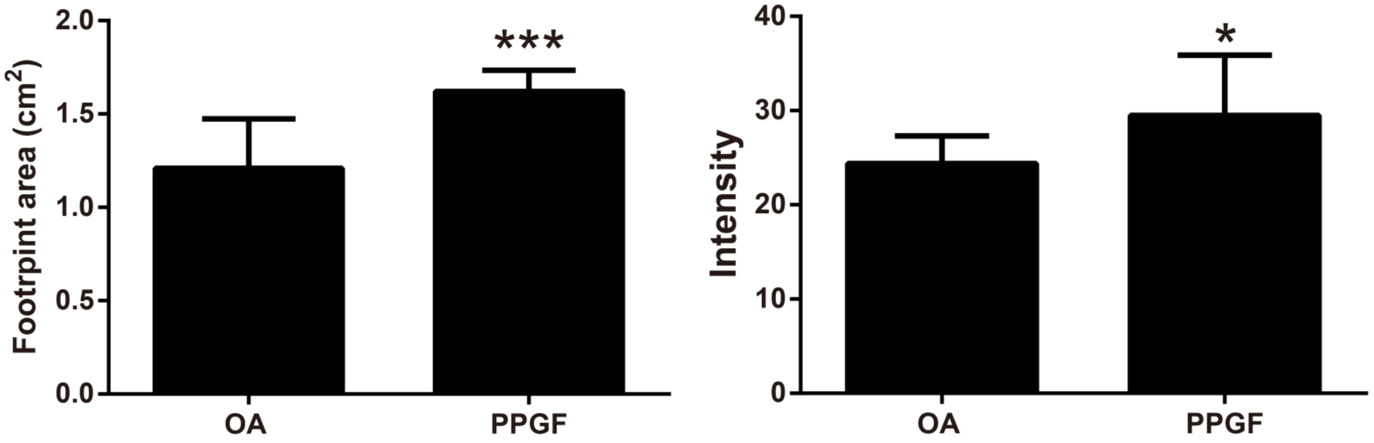

Supplementary Fig. 4 Footprint area and intensity afer therapy for 4 weeks were recorded and analyzed by VisuGait software (Mean ± SD, n=5; *means p<0.05, *** means p<0.001).
