# Supplementary material for: ROS-responsive PPGF nanofiber membrane as a drug delivery system for long-term drug release in attenuation of osteoarthritis
Source: NPJ Regen Med. 2022 Nov 3;7:66. doi: 10.1038/s41536-022-00254-3 (PMC9630282; doi:10.1038/s41536-022-00254-3)
Supplement: Supplementary file 2 — REPORTING SUMMARY [file 41536_2022_254_MOESM2_ESM.pdf]

nature portfolio

Corresponding author(s): Dr Zhenhui Lu

Last updated by author(s): Aug 4, 2022

Reporting Summary

Nature Portfolio wishes to improve the reproducibility of the work that we publish. This form provides structure for consistency and transparency in reporting. For further information on Nature Portfolio policies, see our Editorial Policies and the Editorial Policy Checklist.

Statistics

For all statistical analyses, confirm that the following items are present in the figure legend, table legend, main text, or Methods section.

n/a	Confirmed	
☐	☒	The exact sample size (*n*) for each experimental group/condition, given as a discrete number and unit of measurement
☐	☒	A statement on whether measurements were taken from distinct samples or whether the same sample was measured repeatedly
☐	☒	The statistical test(s) used AND whether they are one- or two-sided<br>*Only common tests should be described solely by name; describe more complex techniques in the Methods section.*
☒	☐	A description of all covariates tested
☒	☐	A description of any assumptions or corrections, such as tests of normality and adjustment for multiple comparisons
☒	☐	A full description of the statistical parameters including central tendency (e.g. means) or other basic estimates (e.g. regression coefficient) AND variation (e.g. standard deviation) or associated estimates of uncertainty (e.g. confidence intervals)
☒	☐	For null hypothesis testing, the test statistic (e.g. *F*, *t*, *r*) with confidence intervals, effect sizes, degrees of freedom and *P* value noted<br>*Give P values as exact values whenever suitable.*
☒	☐	For Bayesian analysis, information on the choice of priors and Markov chain Monte Carlo settings
☒	☐	For hierarchical and complex designs, identification of the appropriate level for tests and full reporting of outcomes
☒	☐	Estimates of effect sizes (e.g. Cohen's *d*, Pearson's *r*), indicating how they were calculated

Our web collection on statistics for biologists contains articles on many of the points above.

Software and code

Policy information about availability of computer code

Data collection	None
Data analysis	None

For manuscripts utilizing custom algorithms or software that are central to the research but not yet described in published literature, software must be made available to editors and reviewers. We strongly encourage code deposition in a community repository (e.g. GitHub). See the Nature Portfolio guidelines for submitting code & software for further information.

Data

Policy information about availability of data

All manuscripts must include a data availability statement. This statement should provide the following information, where applicable:

- Accession codes, unique identifiers, or web links for publicly available datasets
- A description of any restrictions on data availability
- For clinical datasets or third party data, please ensure that the statement adheres to our policy

Provide your data availability statement here.

Human research participants

Policy information about studies involving human research participants and Sex and Gender in Research.

Reporting on sex and gender	None
Population characteristics	None
Recruitment	None
Ethics oversight	None

Note that full information on the approval of the study protocol must also be provided in the manuscript.

Field-specific reporting

Please select the one below that is the best fit for your research. If you are not sure, read the appropriate sections before making your selection.

☒ Life sciences ☐ Behavioural & social sciences ☐ Ecological, evolutionary & environmental sciences

For a reference copy of the document with all sections, see nature.com/documents/nr-reporting-summary-flat.pdf

Life sciences study design

All studies must disclose on these points even when the disclosure is negative.

Sample size	The sample sizes were chosen according to the literature reports.
Data exclusions	None
Replication	All attempts at replication were successful.
Randomization	The samples/organisms/participants were allocated into experimental groups using random number table.
Blinding	The investigators were blinded to group allocation during data collection and /or analysis.

Reporting for specific materials, systems and methods

We require information from authors about some types of materials, experimental systems and methods used in many studies. Here, indicate whether each material, system or method listed is relevant to your study. If you are not sure if a list item applies to your research, read the appropriate section before selecting a response.

Materials & experimental systems

n/a	Involved in the study	
☐	☒	Antibodies
☒	☐	Eukaryotic cell lines
☒	☐	Palaeontology and archaeology
☐	☒	Animals and other organisms
☒	☐	Clinical data
☒	☐	Dual use research of concern

Methods

n/a	Involved in the study	
☒	☐	ChIP-seq
☐	☒	Flow cytometry
☒	☐	MRI-based neuroimaging

Antibodies

Antibodies used	Antibodies used: describe all antibodies used in the study; as applicable, provide supplier name, catalog number, clone number, and lot number.<br>Primary anti-bodies against MMP-13 (BA2204, Boster, Wuhan, China), TNF-α (bs-10802R, Bioss, Peking, China), IL-6 (BA4339, Boster, Wuhan, China), SOD1 (bs-1079R, Bioss, Peking, China), GSH (bs-11756R, Bioss, Peking, China) and NRF2 (66504-1-Ig, Proteintech, Wuhan, China) were used.

Validation

Describe the validation of each primary antibody for the species and application, noting any validation statements on the manufacturer's website, relevant citations, antibody profiles in online databases, or data provided in the manuscript.

Animals and other research organisms

Policy information about studies involving animals; ARRIVE guidelines recommended for reporting animal research, and Sex and Gender in Research

Laboratory animals	SD rats 3-7 days old were used for Chondrocytes isolation and 3 months old were used for in vivo study.
Wild animals	None
Reporting on sex	The suckling rat used for chondrocytes isolation is not limited by gender. Only the male rats were used for in vivo study.
Field-collected samples	At the end of the experiments, the samples of total joints of the rats were collected by injecting overdose of anesthesia.
Ethics oversight	All experiments involved with animals were performed following the Animal Ethics Committee standards of the Guangxi Medical University.

Note that full information on the approval of the study protocol must also be provided in the manuscript.

Flow Cytometry

Plots

Confirm that:

☒ The axis labels state the marker and fluorochrome used (e.g. CD4-FITC).

☒ The axis scales are clearly visible. Include numbers along axes only for bottom left plot of group (a 'group' is an analysis of identical markers).

☒ All plots are contour plots with outliers or pseudocolor plots.

☒ A numerical value for number of cells or percentage (with statistics) is provided.

Methodology

Sample preparation	Chondrocytes were obtained from SD rats. Cells were washed by PBS and the incubated with serum medium containing 10 nmol/L of fluorescent 2, 7-dichlorodihydrofluorescein diacetate kit (DCFH-DA; Beyotime, Shanghai, China) at 37°C for 30 min. After washed with PBS, the intracellular ROS were detected by a Flow Cytometer.
Instrument	Data collection was used a Flow Cytometer (BD Calibur).
Software	CellQuest was used to collect and analyze the flow cytometry data.
Cell population abundance	Cell population used for Flow Cytometry is 10*6/mL. The purities of the samples are 97.33 %, 98.19 %, 98.19 %, 98.95 % and 98.35 % for P, PF, PGF, PPF and PPGF, respectively.
Gating strategy	In the scatterplot of FSC vs SSC, circle the main cell population R1 to be analyzed, thereby excluding cell debris and noise signals caused by laser background from the analysis area. The blank cells unlabeled with the fluorescent probe were used as a control to set a gate, and the cell group R2 with green fluorescence was circled.

☐ Tick this box to confirm that a figure exemplifying the gating strategy is provided in the Supplementary Information.
